# Towards a Zero-Waste Biorefinery Using Edible Oils as Solvents for the Green Extraction of Volatile and Non-Volatile Bioactive Compounds from Rosemary

**DOI:** 10.3390/antiox8050140

**Published:** 2019-05-21

**Authors:** Ying Li, Kunnitee Bundeesomchok, Njara Rakotomanomana, Anne-Sylvie Fabiano-Tixier, Romain Bott, Yong Wang, Farid Chemat

**Affiliations:** 1 JNU-UPM International Joint Laboratory on Plant Oil Processing and Safety, Guangdong Engineering Technology Research Center for Cereal and Oil Byproduct Biorefinery, Department of Food Science and Engineering, Jinan University, Guangzhou 510632, China; twyong@jnu.edu.cn; 2 GREEN Extraction Team, Université d’Avignon et des Pays de Vaucluse, INRA, UMR408, F-84000 Avignon, France; purepharm@hotmail.com (K.B.); njara.rakotomanomana@univ-avignon.fr (N.R.); anne-sylvie.fabiano@univ-avignon.fr (A.-S.F.-T.); 3 Department of Pharmacognosy and Pharmaceutical Botany, Faculty of Pharmaceutical Sciences, Prince of Songkla University, Hat-Yai, Songkhla 90112, Thailand; 4 INRA, UMR408, Securité et Qualité des Produits d’Origine Végétale, F-84000 Avignon, France; Romain.Bott@paca.inra.fr

**Keywords:** green oleo-extraction, zero-waste biorefinery, natural antioxidants and flavors, food-grade solvents, vegetable oils and derivatives, relative solubility simulation

## Abstract

The zero-waste biorefinery concept inspired a green oleo-extraction of both natural volatile (e.g., borneol, camphor, *o*-cymene, eucalyptol, limonene, α-pinene, and terpinen-4-ol) and non-volatile (e.g., carnosol, carnosic, and rosmarinic acid) bioactive compounds from rosemary leaves with vegetable oils and their amphiphilic derivatives as simple food-grade solvents. It is noteworthy that soybean oil could obtain the highest total phenolic compounds (TPCs) among 12 refined oils including grapeseed, rapeseed, peanut, sunflower, olive, avocado, almond, apricot, corn, wheat germ, and hazelnut oils. Furthermore, the addition of oil derivatives to soybean oils, such as glyceryl monooleate (GMO), glyceryl monostearate (GMS), diglycerides, and soy lecithin in particular, could not only significantly enhance the oleo-extraction of non-volatile antioxidants by 66.7% approximately, but also help to remarkably improve the solvation of volatile aroma compounds (VACs) by 16% in refined soybean oils. These experimental results were in good consistency with their relative solubilities predicted by the more sophisticated COSMO-RS (COnductor like Screening MOdel for Real Solvents) simulation. This simple procedure of using vegetable oils and their derivatives as bio-based solvents for simultaneously improving the extraction yield of natural antioxidants and flavors from rosemary showed its great potential in up-scaling with the integration of green techniques (ultrasound, microwave, etc.) for zero-waste biorefinery from biomass waste to high value-added extracts in future functional food and cosmetic applications.

## 1. Introduction

The large quantities of biomass wastes generated from food processing industries worldwide could be considered as a huge potential resource with high-value compounds for biorefinery, which can provide bio-based chemicals and renewable energy with high added value rather than environmental pollution compromising the economy, environment, and human society in the future [1,2]. Although the existing biorefinery technologies (anaerobic digestion, fermentation, hydrothermal conversion, pyrolysis, etc.) have been widely studied and applied in biomass waste reutilization, increasing concern about high setup costs accompanied by incomplete resource utilization, and even secondary pollution, is compelling the scientific, industrial, and government communities to focus more efforts on developing green biorefinery technologies that integrate innovative technologies, which is crucial in achieving maximum recognition of biowastes as chemical and energy resources [3].

Extraction, as the key step in many common process industries and the major expenditure (≥50%) of the total costs, has been paramount within the scope of biorefinery technological development in particular [4]. Considering the increasing demand to reduce the amount of time, energy, costs, and solvents involved in conventional extraction procedures, green extraction with its principles regarding renewable raw materials, alternative solvents [5,6] reduced energy consumption, process intensification, and production of co-products instead of wastes may be well suited as an alternative for a green biorefinery [7]. Currently, several representative technologies such as ultrasound, microwave, supercritical fluids, and instantaneous controlled decompression have successfully proved their effectiveness for the green extraction of natural products from laboratorial to industrial scale [8,9,10,11,12]. However, only a few studies had achieved a biowaste biorefinery according to the green extraction concept and principles.

Plants, especially herbs and spices, have been the major sources for the extraction of numerous natural products with bioactive properties [13]. The reference matrix chosen for this study is rosemary, a well-known ornamental and aromatic plant that has been cultivated along the Mediterranean Sea and widely applied for different purposes. It contains phenolic compounds of great interest for their high antioxidant activities, which are attributed to known carnosol, carnosic, and rosmarinic acids as main constituents [14,15,16]. After the production of rosemary essential oils, the natural phenolic compounds in the residual rosemary have still gained growing interest for direct extraction. Although water or its ethanolic mixture have been recognized as green solvents for extracting rosemary in a sustainable and safe manner, the low extraction yield and unsuitability for the extraction of maximal bioactive compounds other than a single volatile or non-volatile compound limit its application [17,18]. Recently, the application of vegetable oils as alternative solvents for extracting bioactive compounds in various fields has been revisited. These non-polar edible solvents have the advantage of being non-toxic, non-volatile, and non-irritating. Moreover, the endogenous micronutrients like phospholipids, sterols, monoglycerides, and diglycerides are not handicaps for food, cosmetic, or nutraceutical applications [19]. Considering the polarity of oil constituents and the previous experience, it is believed that such bio-based solvents could also be used for the total valorization of rosemary based on the biorefinery and green extraction concept [20,21,22].

**Figure 1:**
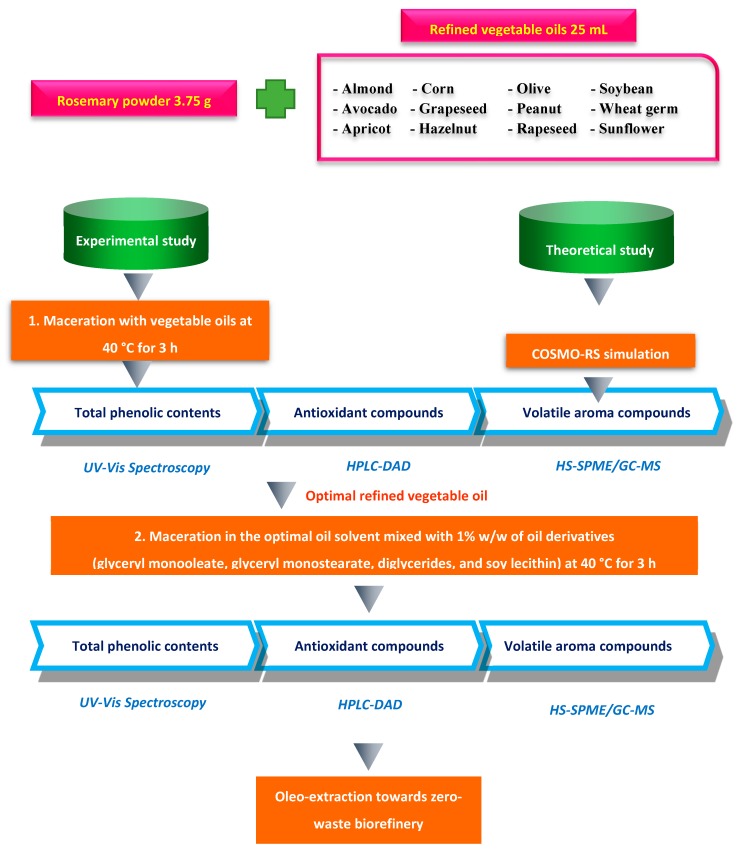
Schematic experimental design. UV-Vis: Ultraviolet-visible; HPLC-DAD: High performance liquid chromatography- diode array detector; HS-SPME: Head space solid-phase microextraction; GC-MS: Gas chromatography-mass spectroscopy

The novelty of this work is the development of a direct oleo-extraction method towards the zero-waste biorefinery concept using vegetable oils and their amphiphilic derivatives as solvents, which facilitates the simultaneous extraction of both volatile and non-volatile bioactive compounds from rosemary leaves (Figure 1). The large consumption of conventional volatile organic solvents was replaced in this maximal extraction of rosemary compounds so that potential risk factors could be avoided. Moreover, the COSMO-RS (COnductor like Screening MOdel for Real Solvents) simulation study helped to theoretically predict the solvent–solute miscibility and further confirm the experimental extractability of all oily solvents for a better comprehension of the dissolving mechanism. In addition, the maximal extraction efficiency of refined soybean oils was verified for the first time with the addition of their amphiphilic derivatives as food-grade surfactants in both a theoretical and an experimental way.

## 2. Materials and Methods

### 2.1. Plant Material and Chemicals

Dried rosemary (*Rosmarinus officinalis* L.) leaves after deodorization were provided by Naturex, Avignon, France. For extraction solvents, refined corn, olive, avocado, wheat germ, hazelnut, apricot, sweet almond, and soybean oils were obtained from *ieS* LABO, Oraison, France. Refined peanut, sunflower, and rapeseed oils were obtained from Auchan, Avignon, France. Refined grapeseed oil was obtained from Tramier, Marseille, France. Glyceryl monooleate (GMO), glyceryl monostearate (GMS), diglycerides (Geleol™, Gattefosse), and soy lecithin were obtained from Oleos, Lunel, France. For analysis, solvents of analytical grade including methanol (99.8%), acetonitrile (99.8%), n-hexane (99.5%), phosphoric acid (85%), trifluoroacetic acid (99.8%), sodium bicarbonate (99.7%), and Folin–Ciocalteu’s reagent, and water of HPLC (high-performance liquid chromatography) grade as well, were purchased from VWR Chemicals, Darmstadt, Germany. Standards of carnosol (purity 98%), carnosic, and rosmarinic acid (purity 97%) used in the HPLC analysis were purchased from Sigma-Aldrich, Munich, Germany.

### 2.2. Fatty Acid Methyl Ester (FAME) Analysis

The fatty acid composition of various vegetable oil solvents was determined according to our developed FAME method [23]. FAMEs were obtained after the methylation of oil samples, from which the supernatants were transferred into vials for further GC-FID (Gas chromatography- flame ionization detector) analysis by a 7820A GC system (Agilent Technologies, Palo Alto, CA, USA) equipped with an FID detector and auto-sampler using a BD-EN14103 capillary column (30 m × 0.32 mm × 0.25 µm) with helium as a carrier gas at the linear velocity of 35 cm/s and glyceryl triheptadecanoate (C_54_H_104_O_6_) as the internal standard. One microliter of sample was injected in split mode (split ratio 1:20) at 250 °C. The oven temperature program was operated as follows: initial temperature at 50 °C, increasing at 20 °C/min to 180 °C and at 2 °C/min from 180 °C to 230 °C, held isothermally at 230 °C for 10 min. Data were collected using Agilent EZChrom Elite (Palo Alto, CA, USA) software and fatty acids were identified by referring to 37 FAME standards (Supelco, Bellefonte, PA, USA). FAMEs were quantified as the relative percentage of the total fatty acids.

### 2.3. Solid–Liquid Extraction

Solid–liquid extractions were referred to a previous study [6]. Briefly, the dried rosemary leaves were mechanically ground and passed through No. 60 mesh screens (0.25 mm) for further maceration. The resulting fine powders (3.75 g) were poured into flasks containing 25 mL of various vegetable oils on a magnetic stirrer plate (RT-10, IKAMAG, Staufen, Germany) at 40 °C for 3 h. Meanwhile, the total phenolic content (TPC) in the rosemary leaves was determined by reflux extraction (EMA0250 Thermo Fisher Scientific, Waltham, MA, USA) of rosemary leaf powders (3.75 g) in 25 mL of aqueous methanol (methanol/water, 90:10 *v/v*) at the boiling point for 30 min. All samples were then centrifuged at 2739× *g* for 15 min at 4 °C in a refrigerated centrifuge (4–16k, Sigma-Aldrich, Munich, Germany). A modified liquid–liquid extraction procedure was used to obtain the phenolic extracts in vegetable oils, where 10 mL of vegetable oils were mixed with n-hexane (1:1, *v/v*) and then extracted with 20 mL of aqueous methanol (methanol/water, 60:40 *v/v*) thrice. The extracts were combined, washed with n-hexane, and then filtered through filters (0.45 μm). All experiments were carried out in triplicate. The methanolic extracts were evaporated to 5 mL and stored at −18 °C for subsequent analyses.

### 2.4. Folin–Ciocalteu Assay

Folin–Ciocalteu assay was used to determine the TPC in the oily extracts. Folin–Ciocalteu’s reagent in 500 µL of water (20%, *v*/*v*) was mixed with 50 µL of methanolic extracts before adding 1 mL of NaHCO₃ solution (10%) and then placed in the dark for 30 min. The absorbance was measured at 760 nm against the blank using a UV-Vis spectrometer (Biochrom Libra S22, Cambridge, UK). The TPC measurements were performed in triplicate and the results were reported as milligrams of rosmarinic acid equivalent per 3.75 g of dried materials.

### 2.5. HPLC Analysis

Carnosol, carnosic, and rosmarinic acids were quantitatively analyzed using an HPLC system (Agilent 1100, Les Ulis, France) equipped with a photo diode array detector (DAD) according to our internal method developed [9], whose procedures are detailed below. Carnosol and carnosic acid were detected at a wavelength of 230 nm using a C18 column (1.8 µm, 4.6 mm × 50 mm, Zorbax Eclipse XBD-C18, Agilent Technologies, Courtaboeuf, France). The mobile phase was isocratic and composed of 0.5% phosphoric acid in water:acetonitrile (35:65, *v/v*). Samples (5 µL) were injected with a flow rate of 1.5 mL/min and the column oven temperature was 25 °C. Rosmarinic acid was detected at a wavelength of 328 nm using a C18 column (5 µm, 4.6 mm × 250 mm, Zorbax SB, Agilent Technologies, Courtaboeuf, France). The mobile phase composed of acetonitrile (32%) and 0.1% of trifluoroacetic acid in water (68%). Samples (5 µL) were injected with a flow rate of 1 mL/min and the column oven temperature was 20 °C with a 10 min runtime.

### 2.6. Headspace Volatile Analysis

The volatile aroma compounds (VACs) in oily rosemary extracts were identified and quantified by headspace solid-phase microextraction (HS-SPME) coupled to gas chromatography mass spectrometry (GC/MS-QP2010, Shimadzu, Kyoto, Japan) with an AOC 5000 auto-injector (Shimadzu, Kyoto, Japan) [24]. The auto sampler was operated in SPME mode using a divinylbenzene-carboxen-polydimethylsiloxane fiber (2 cm, 23 gauge, 50/30 µm DVB/CAR/PDMS; Supelco, Bellefonte, PA, USA) for extraction. For each extraction, oily extract (4 g) was hermetically sealed in screw-top vials (20 mL) containing aluminum seals and PTFE/silicone septa from Grace, France. The samples were equilibrated during incubation time at 35 °C for 15 min before the automatic insertion of the SPME device into the sealed vial, where the fiber was exposed to the headspace of samples at the same temperature for 25 min. The samples were agitated during the incubation and extraction procedures, and the SPME fiber was subsequently removed and inserted into the GC-MS (Gas chromatography-mass spectroscopy) injector port for desorption at 250 °C for 5 min.

The GC-MS analysis was performed using a QP2010 (Shimadzu, Kyoto, Japan) equipped with a capillary column UB-WAX (30 m × 0.25 mm × 0.5 µm) and helium as a carrier gas at the constant flow of 35 cm/s. The initial oven temperature of 35 °C was held for 2 min, then rose at 5 °C/min until 230 °C. The temperature of the transfer line connecting the GC-MS was held at 250 °C. The inlet was operated in the splitless mode and the mass spectrometer operated in the electron impact mode at 70 eV with continuous scans (every 0.2 s) over the mass-to-charge ratio (*m/z*) from 35 to 250. Data were collected using GCMSsolution software 2.40 (Shimadzu, Kyoto, Japan) and the major VACs were identified as compared to their linear retention index and mass spectra with those of authentic standards, as well as with the NIST’98 (US National Institute of Standards and Technology (NIST), Gaithersburg, MD, USA) mass spectral database.

### 2.7. In Silico Solubility Study: COSMO-RS

The in silico solubility of main antioxidant compounds (carnosol, carnosic, and rosmarinic acid) and VACs (eucalyptol, camphor, α-pinene, borneol, *o*-cymene, limonene, and terpinen-4-ol) from rosemary in the mixture of optimal oil solvent and its derivatives was studied by the more sophisticated COSMO-RS software package from COSMO*logic*, Leverkusen, Germany. The chemical structure of all solvents and solutes studied was mutually transformed by JChemPaint version 3.3 (GitHub Pages, San Francisco, CA, USA) into the simplified molecular input line entry syntax (SMILES) notations, which could be further simulated to their three-dimensional σ-surface modelling (Figure S1, Supplementary Materials) by embedded Turbomole (TmoleX, version 7.1), where green to yellow codes the weakly polar surfaces, blue represents electron-deficient regions (δ^+^), and red codes electron-rich regions (δ^−^). This 3D information on the repartition of charge density on the molecular surface can be reduced to σ-profiles and σ-potentials, which could be used for subsequent prediction of the interactions between oily solvents and bioactive compounds based on a quantum-chemical approach. COSMO-RS is known as a modern and powerful method with the combination of quantum chemical considerations (COSMO) and statistical thermodynamics (RS) for molecular description with predicted thermodynamic properties and further solvent prescreening without any experimental data [18].

**Figure 2:**
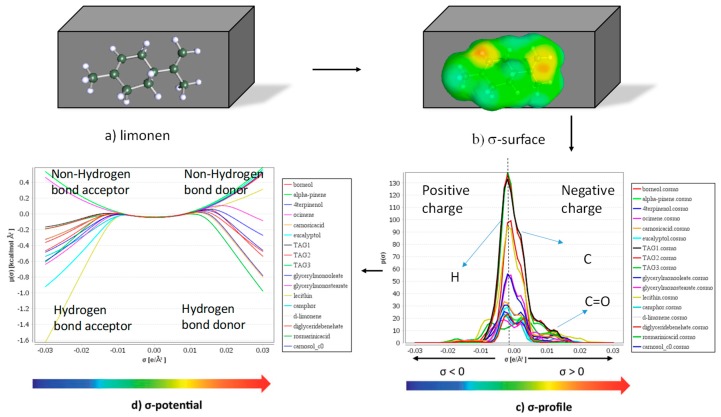
Schematic COSMO-RS (COnductor like Screening MOdel for Real Solvents) step-wise procedures.

As depicted in Figure 2, COSMO-RS generally involves two major steps. All molecules are embedded into virtual conductors simulated in the first step by the COSMO model, where the molecule induces a polarization charge density (σ) on its surface that is a good local descriptor of the molecular surface polarity. Therefore, molecules during the quantum calculation are converged to their energetically optimal states in the conductor with respect to electron density and geometry. Density functional theory (DFT) with triple zeta valence polarized basis set (TZVP) were used as the standard quantum chemical method throughout this study.

The statistical thermodynamics calculation was used in the second step. This polarization charge density was used for the quantification of the interaction energy of pair-wise interacting surface segments concerning electrostatics and hydrogen bonding. The 3D distribution of the polarization charges on the surface of each molecule was converted into a surface composition function (σ-profile) that provided the information about molecular polarity distribution. The thermodynamics of molecular interactions based on the σ-profile obtained was then calculated to chemical potential of the surface segment (σ-potential) using the COSMO*thermX* program (version C30 release 13.01). The σ-potential describes the likeliness of the solute compound interacting with solvents according to their polarities and hydrogen bonds, where the part of the negative charge of molecules (hydrogen bond acceptor) was located on the right side with positive σ-profile values, whereas the part of the positive charge (hydrogen bond donor) was located on the left side with negative σ-profile values. The solvent’s affinity for polarity surface could be interpreted according to the σ-profile and σ-potential in order to better understand the solute–solvent interaction in a mixture state.

In addition, COSMO*thermX* also calculated the relative solubility of COSMO-RS simulated volatile and non-volatile bioactive compounds in various oil solvents and mixtures with their derivatives based on the logarithm of the solubility in mole fractions. The relative solubility *x_j_* of compound *j* is always calculated in infinite dilution according to the equation as follows:

(1) $$\log_{10}(x_{j})=\log_{10}\left[\frac{exp(\mu_{j}^{pure}-\mu_{j}^{solvent}-△G_{j.fusion})}{RT}\right],$$

where $\mu_{j}^{pure}$ is the chemical potential of pure compound j (J/mol), $\mu_{j}^{solvent}$ is the chemical potential of j at infinite dilution (J/mol), $△G_{j.fusion}$ is the free energy of fusion of j (J/mol), $x_{j\;}$ is the solubility of j (g/g solvent), R is the gas constant, and T is the temperature (K).

The logarithm of the best solubility is set to 0, and all other solvents were given referring to the best solvent. The solubility calculation of major antioxidants and VACs in vegetable oils and mixtures with their derivatives was all performed at 40 °C. A solvent with a log10($x_{j}$) value of −1.00 yields a solubility which is decreased by a factor 10 compared to the best solvent.

### 2.8. Statistical Analysis

The experimental solubility of bioactive compounds extracted in 16 oily solvents was further transformed using MATLAB 2015a (The MathWorks, Inc., Natick, MA, USA). First, principal component analysis (PCA) reduced the dimensionality of our data set (individuals: oily solvents; variables: TPC, antioxidants, and VACs) by linear combination into new coordinate systems. The solute concentrations in 16 oily solvent systems were taken for the principal component determination so as to compare the solubility of these 16 solvents in PCA plotting graphs. Clustering analysis was used to classify the closest individuals into clusters, where Ward’s hierarchical clustering was applied to calculate dissimilarities from the Euclidean distances and aggregation criterion corresponding to the minimization of the within-cluster inertia and the maximization of between-cluster inertia. Concerning the extractability of non-volatile and volatile bioactive compounds in different solvents, this statistical method could partition oily solvents into homogeneous clusters with a low within-variability, which are different from others with a high between-variability. Given this, a dendrogram was made to illustrate the aggregations made at each successive stage.

## 3. Results

### 3.1. Composition of Vegetable Oils

As shown in Table 1, the fatty acid composition of different vegetable oils was affected by the nature and origin [16]. According to the main three fatty acid chains on the triglyceride (TAG), it was noted that most vegetable oils (rapeseed, hazelnut, sweet almond, apricot, sunflower, and peanut oils) contained the same major fatty acids (e.g., oleic acid–oleic acid–linoleic acid (OOL)) with different proportions. Similarly, the major fatty acid compositions in grapeseed, corn, wheat germ, and soybean oils were oleic acid–linoleic acid–linoleic acid (OLL) and avocado oil had its own main fatty acid composition as oleic acid–oleic acid–palmitic acid (OOP). Moreover, three oleic acids (OOO) was inferred in the TAG of olive oil. The above-mentioned four categories of three major fatty acids represented 12 vegetable oils, which could be selected to constitute TAG structures for further computational simulations. Apart from the major composition in oils, it must be mentioned that minor compounds including diglycerides (DAGs), monoglycerides (MAGs), and phospholipids could be considered as food-grade surfactants, which can strongly affect the physiochemical and dissolving properties of the vegetable oils [25].

### 3.2. Experimental Dissolving Power of Refined Vegetable Oils and Mixtures with Their Derivatives

#### 3.2.1. Extractability of Total Phenolic Compounds

**Figure 3:**
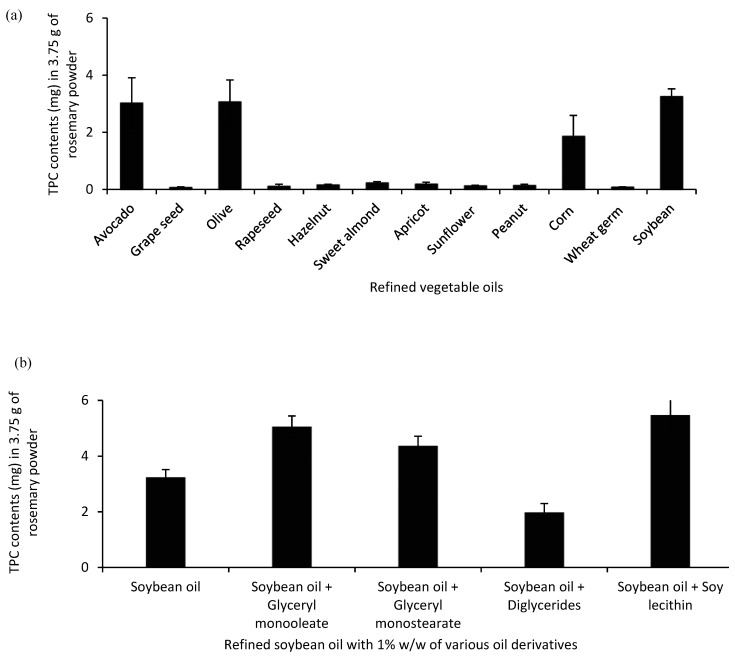
Total phenolic content (TPC) in rosemary oleo-extracts by using (**a**) refined vegetable oils and (**b**) refined soybean oil with the addition of oil derivatives as solvents.

The TPC in refined vegetable oils and mixtures with their derivatives as solvents quantified by the Folin–Ciocalteu test is illustrated in Figure 3. Generally, these refined oils consisting of more than ≥98% TAGs are often considered as strictly non-polar liquid solvents which are unfavorable for extraction. Therefore, only 1/3 of the refined oil solvents (avocado, olive, corn, and soybean) in this study showed relatively higher TPC yield, among which soybean oil obtained the highest (Figure 3a).

This could be explained by its highest content of polyunsaturated fatty acids, allowing it to give itself a lower viscosity corresponding to a higher diffusivity that helps to increase the extraction yield. However, it was found that there is no obvious correlation between the TPC extraction yield (0.07–3.25 mg in 3.75 g of rosemary powders) and the polyunsaturation level of refined oil solvents (6.2–75.8%), which indicates that the TPC extraction yield may be more related to the presence of endogenous amphiphilic compounds like partial glycerides and phospholipids, and the refining degree of oils as well. For this, with the aim of further improving the dissolving power of refined soybean oils, the influence of oil derivatives on the TPC extraction was studied at the same dosage (1% *w/w*). Compared to the refined soybean oil, the addition of amphiphilic oil derivatives helped to increase the TPC extraction efficiency with the exception of DAG (Figure 3b). The performance of these food additives was found corresponding to the order of their hydrophilicity: soy lecithin > GMO > GMS > DAG. The presence of more polar phosphate groups in the soy lecithin allowed the formation of more reverse micelles containing phenolic compounds in the water pool center [26], which led to the highest TPC increment by approximately a factor of 1.7. Because of the higher lipophilicity of DAG, it obtained a lower TPC yield than MAG. GMO had a higher TPC extraction yield than that of GMS, which could be explained by the fact that the unsaturated GMO with a higher polarity and lower viscosity could facilitate the TPC extraction.

#### 3.2.2. Extractability of Carnosol, Carnosic, and Rosmarinic Acids

**Figure 4:**
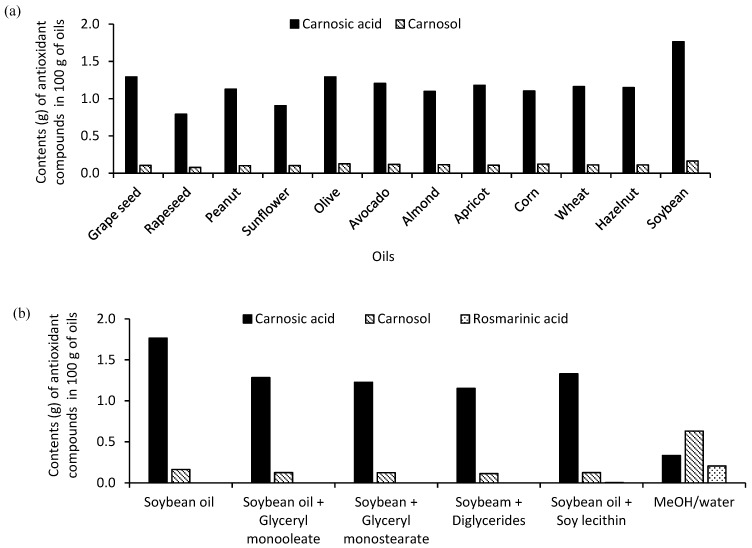
Content of carnosol and of carnosic and rosmarinic acids in rosemary oleo-extracts determined by HPLC using (**a**) refined vegetable oils and (**b**) refined soybean oil with the addition of oil derivatives as solvents.

Specifically, the content of main antioxidants in the refined oil solvents and their mixtures with oil derivatives is presented in Figure 4. All refined vegetable oils used were capable of extracting apolar carnosol and carnosic acid to various degrees, whereas polar rosmarinic acid was undetectable in all oil solvents. Similar to TPC results, the highest content of carnosic acid (1.76 mg in 100 g oil) and carnosol (0.16 mg in 100 g oil) was also observed in refined soybean oils (Figure 4a), which was thus selected for further extraction improvement with the addition of 1% *w/w* of oil derivatives. Compared to aqueous methanolic rosemary extracts, oily solvents could achieve a higher extraction yield of main antioxidants in total, although extracts by organic solvents obtained considerable carnosol and rosmarinic acids (Figure 4b).

Soy lecithin was the most suitable additive for the extraction of main rosemary antioxidants, followed by GMO, GMS, and DAG. Although the content of carnosic acid and carnosol in the solvent system was not as much as that in refined soybean oils, it is interesting to quantitatively detect rosmarinic acids within. Its higher extractability for both polar and non-polar compounds could also be the reason why the solvent system gave the highest TPC content compared to other oily solvents. Considering the relatively high extractability to phenolic compounds, refined soybean oil and its mixture with soy lecithin could be considered as good solvents for the extraction of rosemary antioxidant compounds.

#### 3.2.3. Extractability of Volatile Aroma Compounds (VACs)

The standard of seven VACs at the same different concentration gradients was analyzed first in different aromatized oils under the same HS-SPME/GC-MS conditions in order to evaluate the extraction efficiency of the oily matrix and HS-SPME fiber. The fiber used was kept consistent and updated for analyses of each independent aromatized oil in order to minimize the error quantification. The content of major VACs in all oily solvents is presented in Figure S2, Supplementary Materials.

Major monoterpene hydrocarbons (e.g., α-pinene, limonene, and *o*-cymene) and highly odoriferous oxygenated monoterpenes (e.g., borneol, camphor, eucalyptol, and terpinen-4-ol) were both quantified in all oily extracts, but their contents were different from those in rosemary essential oils [27]. Eucalyptol was found as the major compound in oily solvents with the highest concentration, followed by camphor, α-pinene, *o*-cymene, and limonene. Different refined oil solvents showed an individual difference regarding the proportion of major VACs extracted (Figure S2a, Supplementary Materials). In other words, the highest contents of major VACs observed here were eucalyptol (49.26% *w/w*) in wheat germ oil, camphor (13.48% *w/w*) in rapeseed oil, α-pinene in soybean oil (10.70% *w/w*), terpinen-4-ol (3.65% *w/w*) in corn oil, as well as limonene (7.96% *w*/*w*), *o*-cymene (9.27% *w/w*), and borneol (3.05% *w*/*w*) in hazelnut oil. Furthermore, the mixture of refined soybean oil and 1% w/w of oil derivatives was studied as well to verify its performance consistency (Figure S2b, Supplementary Materials). Compared to refined soybean oil solely, the addition of oil derivatives could help to significantly increase the extraction yield of eucalyptol and α-pinene than other VACs. The highest content of camphor (16.61% *w*/*w*), terpinen-4-ol (4.24% *w*/*w*), and borneol (3.66% *w*/*w*) was found in the mixture of refined soybean oil and DAG. Therefore, it is worthy to note that adding oil derivatives to refined oil solvents could increase the extraction selectivity of VACs, which gave a higher yield of total oxygenated monoterpenes than total monoterpene hydrocarbons compared to using refined oils alone (Figure S2c, Supplementary Materials).

### 3.3. Theoretical Dissolving Power of Refined Soybean Oils and Mixtures with Their Derivatives

From experimental results above, it can be noted that oily solvents with various solutes are multi-component systems for which it is difficult to predict the dissolving power due to their complexities. Conventional solubility simulation methods (Hilderbrand or Hansen solubility parameters, etc.) often underestimate molecular structural difference and interaction force (type, proportion, and position of three main fatty acid chains in TAG, etc.) resulting in predicted results with little differences between each other. Thus, a powerful COSMO-RS simulation was conducted to determine the relative solubility of both phenolic antioxidants and VACs in refined soybean oils and their mixtures with oil derivatives. The σ-potentials of solvents and solutes (i.e., TAGs, oil derivatives, major antioxidants, and VACs) derived from the COSMO-RS simulation can be successfully employed to describe and classify solvents in a purely predictive manner with a good consideration of hydrogen bond donor–acceptor interactions. Generally, the region σ ± 0.01 e/A^2^ is considered as non-polar or weakly polar. For instance, the σ-profile of limonene showed two peaks resulting from the hydrogen atoms on the negative side and from the carbon atoms on the positive side. Therefore, the σ-potential of limonene was similar to the U-shape centered at σ = 0, which is the typical characteristic of non-polar solvents. As represented in Table 2, the TAG composition possibilities in refined soybean oils including 66% of TAG 1 (R1: C18:2, R2: C18:2, R3: C18:2), 23% of TAG 2 (R1: C18:1, R2: C18:2, R3: C18:2), and 11% of TAG 3 (R1: C18:2, R2: C18:1, R3: C16:0), and their mixtures with oil derivatives could be taken into consideration in this quantic chemical approach. As the logarithm of the best solubility is set to 0, all these solvents could be predicted under simulated industrial conditions at 40 °C. It was found that the relative solubility of all components was below zero, indicating that these oily solvents may not be perfect solvents theoretically though they have considerable dissolving power in real extractions. Compared to the relative solubility value of refined soybean oil as the reference solvent, oily solvents with higher relative solubility values (in green) could be recognized as having better dissolving powers. Otherwise, solvents with poorer dissolving capacities had lower relative solubility values (in red). The theoretical solubility of both non-volatile antioxidants and VACs in the mixture of refined soybean oil and oil derivatives was in good consistency with experimental data, with the exception of camphor in refined soybean oils with DAG. Overall, refined soybean oils with soy lecithin (1%, *w*/*w*) could be theoretically considered as the optimal solvent because of its near-zero relative solubility for all bioactive compounds, which is also in good accordance with experimental results obtained previously.

### 3.4. Classification of Oily Solvents

**Figure 5:**
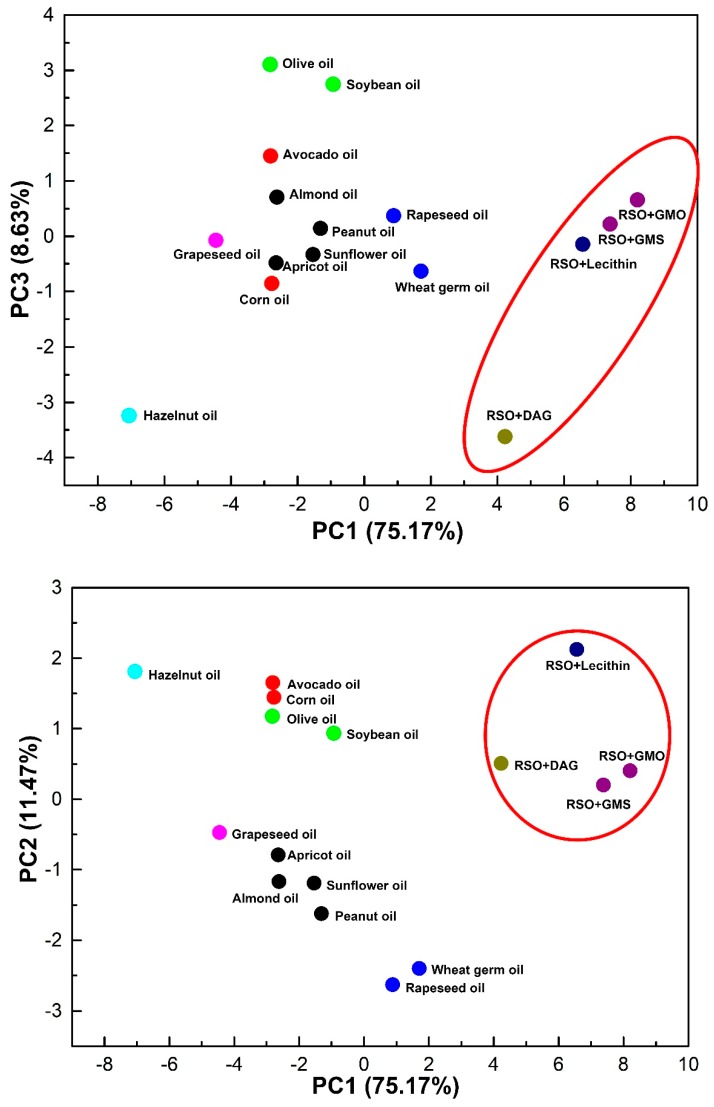
Dissolving power of oily solvents in principal component analytical plots (PC1 *versus* PC2 and PC1 *versus* PC3) corresponding to volatile and non-volatile bioactive compounds extracted.

**Figure 6:**
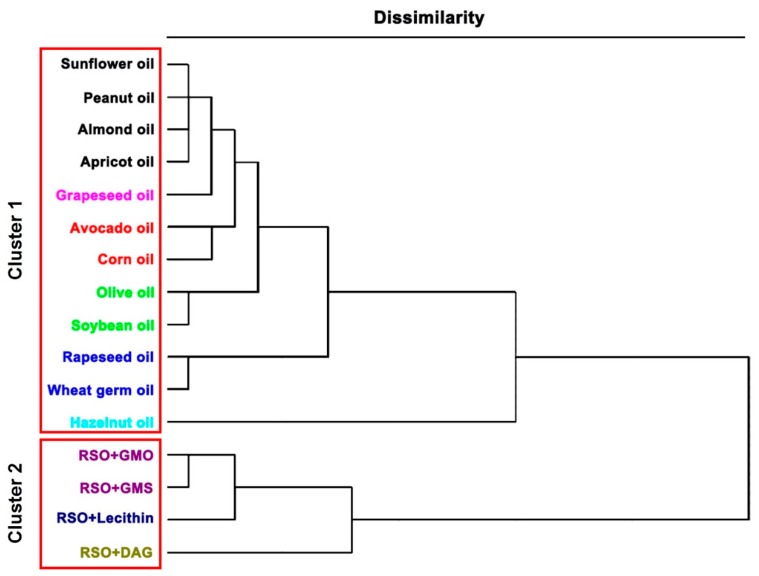
Dendrogram resulting from Ward’s hierarchical cluster analysis corresponding to the dissolving power of oily solvents in terms of volatile and non-volatile bioactive compounds extracted.

Generally, the variability of independent principal components is the percentage of information which is well represented for other components. In this case, the PCA plotted all oily solvents with the main components PC1, PC2, and PC3 representing 95.27% of the original information with less than 5% loss of information (Figure 5). The distribution of 16 oily solvents on the 2D mapping graphs (PC1 versus PC2 and PC1 versus PC3) described well their inner similarity depending on the experimental solubility of TPC, non-volatile antioxidants, and VACs inside, which is directly related to independent variables that can bring significant information for the oily solvent discrimination. The following clustering analysis was carried out on the basis of the concentration of both non-volatile and volatile bioactive compounds as well (Figure 6). The dendrogram showed that the dissolving power of oily solvents could be finally aggregated into two main clusters. The first cluster including all refined vegetable oils showed the highest dissimilarity as compared to the second cluster, which indicated the significant effect of the addition of oil derivatives to refined oil solvents on their extraction improvement.

The oily solvents merging together within each cluster showed low dissimilarity on the dissolving power. Therefore, the low dissimilarity was found in cluster 1 among several sub-clusters in the same color (refined sunflower, peanut, almond, and apricot oils, etc.), and in cluster 2 between refined soybean oil with GMO and GMS. However, some oily solvents presented high dissimilarity corresponding to their lower or higher dissolving powers as compared to others. For instance, refined wheat germ and rapeseed oils in the same sub-cluster had nearly the same dissolving power with much lower yields for all bioactive compounds extracted, although they had a relatively higher affinity to VACs. Refined hazelnut oil with the lowest dissolving power showed the highest dissimilarity, which was isolated at a distance from others in the PCA graphs. A similar case happened in another cluster for refined soybean oil with DAG, which also had the lowest dissolving power. Nonetheless, refined soybean oil with soy lecithin showed a high dissimilarity because of its best performance in the oleo-extraction of both non-volatile and volatile bioactive compounds from rosemary. Interestingly, refined vegetable oils having similar TAG composition were distributed in different sub-clusters, which also further states the fact that the dissolving power of these complex solvents is more correlated to their endogenous amphiphilic constitutes. Refined sunflower oil was previously reported as the optimal product for the extraction of six VACs from basil [24]. Nevertheless, refined soybean oil had a better dissolving power in this work for the maximal extraction of non-volatile antioxidants and VACs from rosemary, indicating the specificity of oleo-extraction that is worthy for case-by-case studies. All results were in good consistency with the PCA observation and theoretical relative solubility results, which further proved the effectiveness of COSMO-RS for a priori solvent screening before trial and error.

Since the dissolving power of vegetable oils have been confirmed as alternative solvents for natural product extractions, a novel oleo-extraction process could be subsequently developed based on green extraction principles with the integration of innovative techniques (ultrasound, microwave, etc.), resulting in a high-efficiency, time- and energy-saving extraction, as well as high value-added lipidic products with the maximal risk reduction of oxidation or degradation thanks to the extracted bioactive compounds within [19,28]. In addition, vegetable oils could also be extracted by other alternative eco-friendly solvents (terpenes, 2-MeTHF, liquefied gas, etc.) to n-hexane, which conceivably led to a truly green extraction process for future industrial applications [29,30,31,32].

## 4. Conclusions

This study aimed at developing a green oleo-extraction of total volatile and non-volatile bioactive compounds from rosemary leaves towards a zero-waste biorefinery concept using vegetable oils and their amphiphilic derivative constitutes as bio-based solvents. The experimental results showed that refined soybean oil performed the best concerning the yield of both major phenolic antioxidants and VACs in rosemary. Moreover, the addition of oil derivatives, soy lecithin in particular, surprisingly improved the extraction efficiency of more polar compounds and VACs on the basis of the original oleo-extraction. Meanwhile, a good consistency with COSMO-RS simulation proved its suitability for modeling these supramolecular solvent systems, which could overcome the limit of conventional solubility methods. Considering theoretical, experimental, and statistical results, refined soybean oil with soy lecithin (1%, w/w) appears to be the most promising and economically viable solvent among all oily solvents tested for the maximal extraction of major antioxidants and VACs from rosemary.

From the practical point of view, COSMO-RS could be conducted as a heuristic tool before real extractions for a low-cost and high-accuracy screening of complex solvents or multicomponent solute–solvent mixtures. As the dissolving power of vegetable oils as bio-based solvents has been gradually proved for solutes with a broader range of polarity, the green oleo-extraction updated in this study is more comprehensive, where no additional separation steps are required after extractions. Revisiting this safe, easy-to-use, and eco-friendly method has produced novel enriched oils, thereby providing a relatively green solution towards the biorefinery of wastes from the processing of nearly all plant materials, depending on the market demand in the cosmetic and agro-food industries.

## Figures and Tables

**Table 1:** Composition of fatty acids in vegetable oils identified by fatty acid methyl ester (FAME) analysis.

Fatty Acid Composition (%) ^a^		Refined Vegetable Oils										
	OOP	OOO	OOL	OOL	OOL	OOL	OOL	OOL	OLL	OLL	OLL	OLL
	Avocado	Olive	Rape Seed	Hazel Nut	Almond	Apricot	Sunflower	Peanut	Grapeseed	Corn	Wheat Germ	Soybean
Palmitic acid (C16:0)	16.5	10.4	4.1	6.2	5.1	5.3	4.4	6.0	6.0	11.1	11.8	10.7
Palmitoleic acid (C16:1)	8.5	0.8	0.2	0.4	0.7	0.8	0.1	0.1	0.1	-	0.2	0.5
Stearic acid (C18:0)	0.6	3.5	-	2.0	2.1	1.1	-	-	3.3	1.6	2.6	2.8
Oleic acid (C18:1)	63.9	78.3	62.5	74.2	66.5	59.8	55.5	73.2	14.4	29.5	29.4	23.2
Linoleic acid (C18:2)	9.5	5.5	19.8	16.4	24.7	28.9	35.3	11.9	75.6	55.8	52.0	55.6
α-Linolenic acid (C18:3)	0.6	0.6	9.7	0.1	0.1	0.1	0.1	0.1	0.1	0.9	-	6.3
Arachidic acid (C20:0)	0.1	0.4	0.3	0.1	-	-	-	2.8		0.4	0.5	0.3
Gondoic acid (C20:1)	-	-	1.3	-	-	-	0.2	2.8	0.2	-	-	-
Gadoleic acid (C20:1)	0.2	0.3	-		-	-	-	-	-	0.3	0.3	0.2
Behenic acid (C22:0)	-	0.1	-	0.2	-	-	-	-	-	0.1	0.2	0.4
Erucic acid (C22:1)	-	-	-	-	-	-	-	0.4	-	-	0.2	-
∑PUFAs	10.1	6.2	29.5	16.5	24.8	29.0	36.4	12.0	75.8	56.7	52	61.9
∑MUFAs	72.6	79.4	64.0	74.6	67.2	60.6	55.8	76.5	14.7	29.8	29.8	23.9
∑SFAs	17.2	14.4	4.4	8.5	7.2	6.4	4.4	8.8	9.5	13.2	15.1	14.2

O: Oleic acid; L: Linoleic acid; P: Palmitic acid; PUFAs: polyunsaturated fatty acids; MUFAs: monounsaturated fatty acids; SFAs: saturated fatty acids. ^a^ Some irrelevant fatty acids are not presented in this table.

**Table 2:** Relative solubility of non-volatile and volatile bioactive compounds in refined soybean oil and its mixture with oil derivatives using the COSMO-RS approach.

Bioactive Compound	Oily Solvents				
	Refined Soybean Oil (RSO) ^a^	Addition of Oil Derivatives (1%, *w*/*w*)			
		RSO + DAG	RSO + GMO	RSO + GMS	RSO + Soy lecithin
**Carnosic acid**	−2.7222	−2.7173	−2.7066	−2.7101	−2.3683
Rosmarinic acid	−2.5669	−2.5584	−2.5396	−2.5480	−1.9897
Carnosol	−1,9426	−1.9408	−1.9358	−1.9370	−1.8102
Borneol	−1.5602	−1.5289	−1.5257	−1.5259	−1.4458
Camphor	−0.7953	−0.7956	−0.7946	−0.7932	−0.7931
*o*-Cymene	−0.2403	−0.2407	−0.2411	−0.2413	−0.2409
Eucalyptol	−1.1891	−1.1889	−1.1869	−1.1819	−1.1885
Limonene	−0.3473	−0.3477	−0.3483	−0.3485	−0.3484
α-pinene	−0.6353	−0.6358	−0.6368	−0.6369	−0.6378
Terpinen-4-ol	−1.3907	−1.3896	−1.3859	−1.3827	−1.3395

^a^ Refined soybean oil: 66% TAG 1 (R1 = C18:2, R2 = C18:2, R3 = C18:2), 23% TAG 2 (R1 = C18:1, R2 = C18:2, R3 = C18:2), 11% TAG 3 (R1 = C18:2, R2 = C18:1, R3 = C16:0); DAG: diglycerides; GMO: glyceryl monooleate; GMS: glyceryl monostearate. Grey: Reference; Green: Better or equivalent than reference; Red: Worse than reference.

## Supplementary Materials

The following are available online at https://www.mdpi.com/2076-3921/8/5/140/s1, Figure S1: Three-dimensional chemical structures and σ–surfaces of both volatile and non-volatile solutes, triglyceride possibilities in refined soybean oils and oil amphiphilic derivatives generated by COSMO-RS, Figure S2: Major volatile aroma compounds (VACs) in rosemary oleo-extracts determined by HS-SPME/GC-MS using (a) refined vegetable oils and (b) refined soybean oil with addition of oil derivatives as solvents, (c) the content of total monoterpenes *versus* total oxygenated monoterpenes in various oily solvent systems.
