# Supplementary material for: Towards a Zero-Waste Biorefinery Using Edible Oils as Solvents for the Green Extraction of Volatile and Non-Volatile Bioactive Compounds from Rosemary
Source: Antioxidants (Basel). 2019 May 21;8(5):140. doi: 10.3390/antiox8050140 (PMC6562763; doi:10.3390/antiox8050140)
Supplement: Supplementary file 1 [file antioxidants-08-00140-s001.pdf]

Supplementary materials

Figure S1: Three-dimensional chemical structures and σ–surfaces of both volatile and non-volatile solutes, triglyceride possibilities in refined soybean oils and oil amphiphilic derivatives generated by COSMO-RS.

Figure S2: Major volatile aroma compounds (VACs) in rosemary oleo-extracts determined by HS-SPME/GC-MS using (a) refined vegetable oils and (b) refined soybean oil with addition of oil derivatives as solvents, (c) the content of total monoterpenes *versus* total oxygenated monoterpenes in various oily solvent systems.

Figure S1.

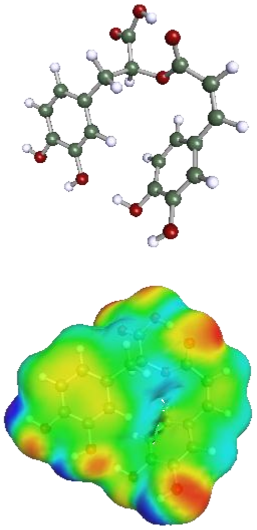

Rosmarinic acid

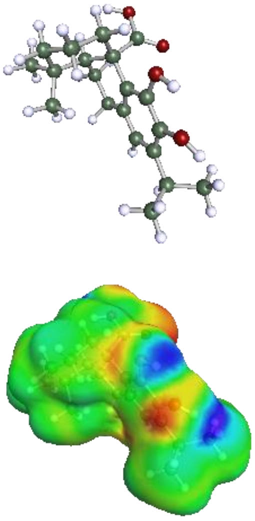

Carnosic acid

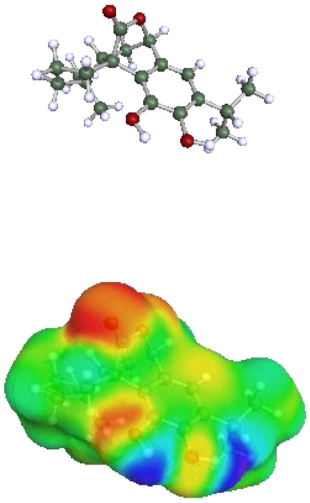

Carnosol

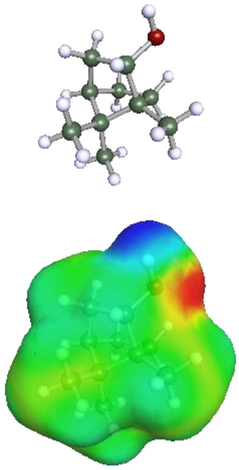

Borneol

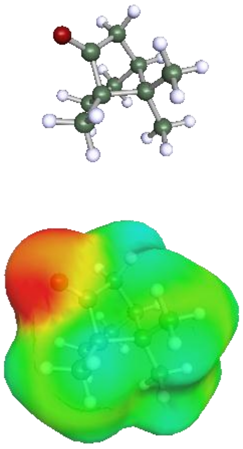

Camphor

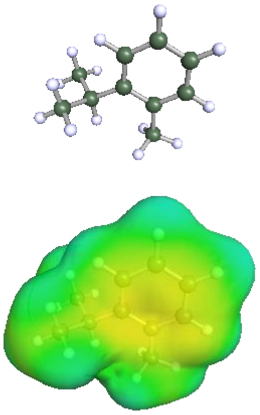

o-cymene

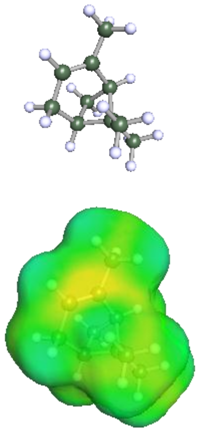

α-pinene

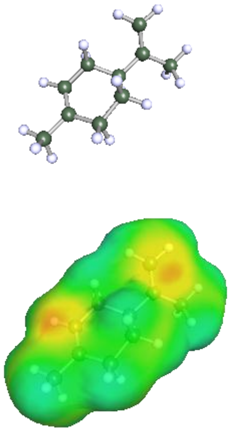

Limonene

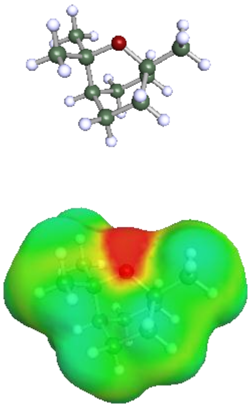

Eucalyptol

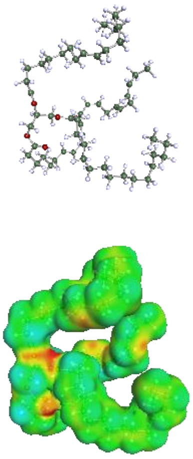

TAG 3

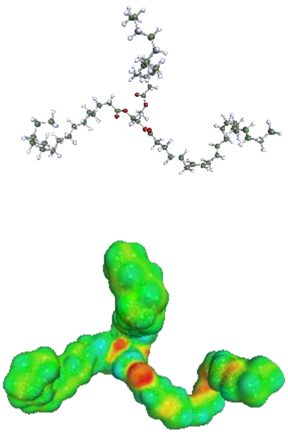

TAG 1

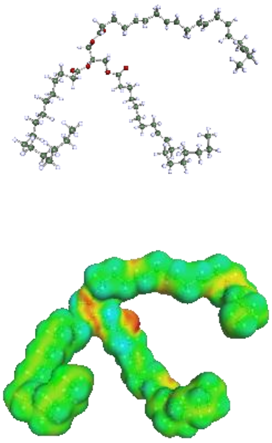

TAG 2

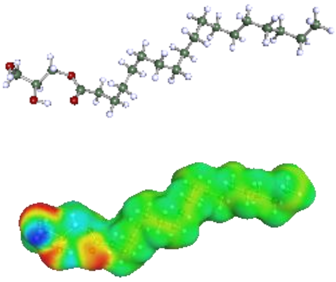

Glyceryl monostearate (GMS)

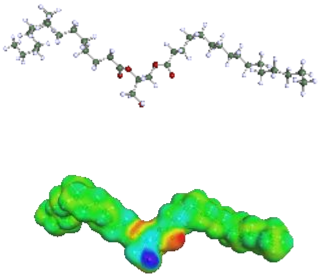

Diglyceride

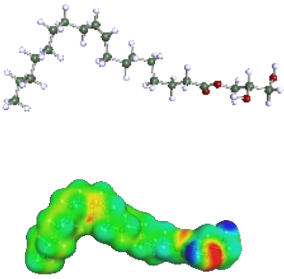

Glyceryl monooleate (GMO)

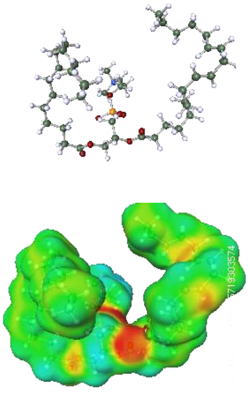

Lecithin

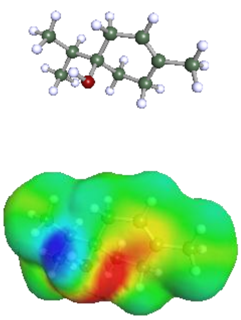

Terpinene-4-ol

Figure S2.

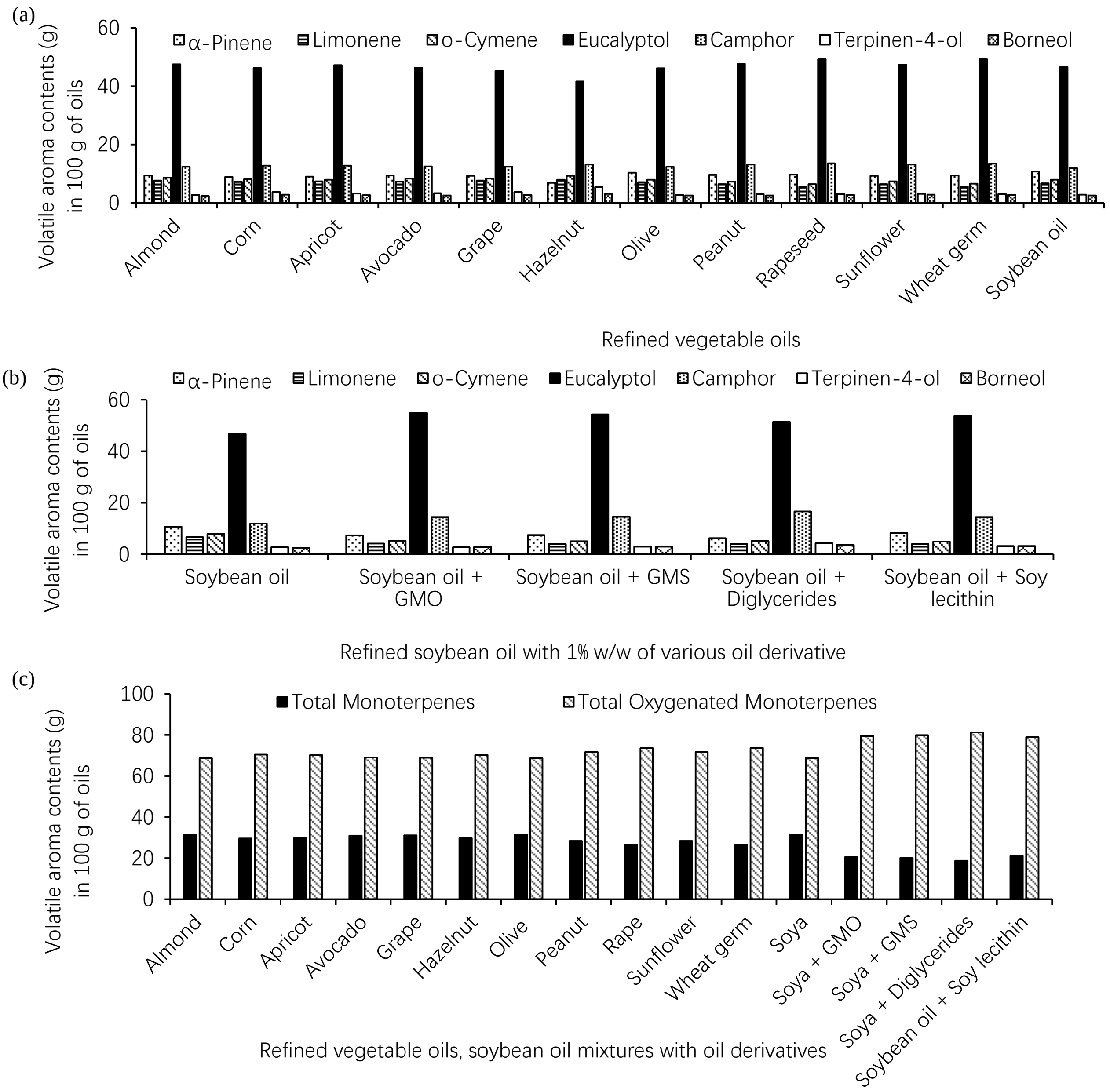
(a)
α-Pinene
Limonene
o-Cymene
Eucalyptol
Camphor
Terpinen-4-ol
Borneol
Volatile aroma contents (g) in 100 g of oils
60
40
20
0
Almond
Corn
Apricot
Avocado
Grape
Hazelnut
Olive
Peanut
Rapeseed
Sunflower
Wheat germ
Soybean oil
Refined vegetable oils
(b)
α-Pinene
Limonene
o-Cymene
Eucalyptol
Camphor
Terpinen-4-ol
Borneol
Volatile aroma contents (g) in 100 g of oils
60
40
20
0
Soybean oil
Soybean oil + GMO
Soybean oil + GMS
Soybean oil + Diglycerides
Soybean oil + Soy lecithin
Refined soybean oil with 1% w/w of various oil derivative
(c)
Total Monoterpenes
Total Oxygenated Monoterpenes
Volatile aroma contents (g) in 100 g of oils
100
80
60
40
20
0
Almond
Corn
Apricot
Avocado
Grape
Hazelnut
Olive
Peanut
Rape
Sunflower
Wheat germ
Soya
Soya + GMO
Soya + GMS
Soya + Diglycerides
Soybean oil + Soy lecithin
Refined vegetable oils, soybean oil mixtures with oil derivatives
